# Interaction of Virstatin with Human Serum Albumin: Spectroscopic Analysis and Molecular Modeling

**DOI:** 10.1371/journal.pone.0037468

**Published:** 2012-05-23

**Authors:** Tanaya Chatterjee, Aritrika Pal, Sucharita Dey, Barun K. Chatterjee, Pinak Chakrabarti

**Affiliations:** 1 Department of Biochemistry, Bose Institute, Kolkata, India; 2 Bioinformatics Centre, Bose Institute, Kolkata, India; 3 Department of Physics, Bose Institute, Kolkata, India; Russian Academy of Sciences, Institute for Biological Instrumentation, Russian Federation

## Abstract

Virstatin is a small molecule that inhibits *Vibrio cholerae* virulence regulation, the causative agent for cholera. Here we report the interaction of virstatin with human serum albumin (HSA) using various biophysical methods. The drug binding was monitored using different isomeric forms of HSA (N form ∼pH 7.2, B form ∼pH 9.0 and F form ∼pH 3.5) by absorption and fluorescence spectroscopy. There is a considerable quenching of the intrinsic fluorescence of HSA on binding the drug. The distance (r) between donor (Trp214 in HSA) and acceptor (virstatin), obtained from Forster-type fluorescence resonance energy transfer (FRET), was found to be 3.05 nm. The ITC data revealed that the binding was an enthalpy-driven process and the binding constants *K*_a_ for N and B isomers were found to be 6.09×10^5 ^M^−1^ and 4.47×10^5^ M^−1^, respectively. The conformational changes of HSA due to the interaction with the drug were investigated from circular dichroism (CD) and Fourier Transform Infrared (FTIR) spectroscopy. For 1∶1 molar ratio of the protein and the drug the far-UV CD spectra showed an increase in α- helicity for all the conformers of HSA, and the protein is stabilized against urea and thermal unfolding. Molecular docking studies revealed possible residues involved in the protein-drug interaction and indicated that virstatin binds to Site I (subdomain IIA), also known as the warfarin binding site.

## Introduction

**Figure 1:**
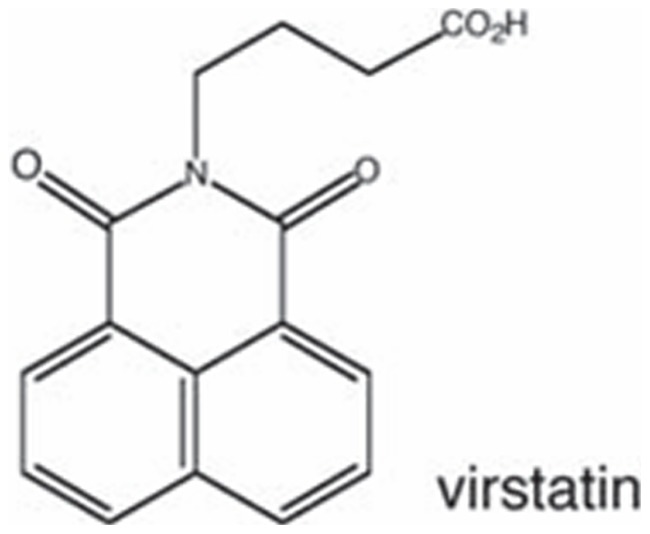
Chemical structure of virstatin.

Chemical genetics is an emerging field of research which employs small molecules to dissect complex biological processes and for studying microbial pathogenesis [1], [2]. In order to dissect the pathogenesis cholera, which still poses a threat to many parts of the world, high throughput screen of 50,000 compounds in small molecule library from Chembridge Research Laboratories was carried out to identify inhibitors of *Vibrio cholerae* virulence factor expression [3]–[6]. Virstatin, 4-[N-(1,8-naphthalimide)]-n-butyric acid (Figure 1), is such a small molecule that attenuates the intestinal colonization of *Vibrio cholerae* by preventing the dimerization of the transcriptional activator ToxT [7], [8]. It also binds to accessory cholera enterotoxin (Ace), an important toxin of *V. cholerae* [9]. In view of this, we planned to carry out the binding studies of virstatin with human serum albumin (HSA), the most abundant protein in the circulatory system. HSA is synthesized in the liver, exported as a non-glycosylated protein and is present in the blood at around 40 mg ml^−1^.

The most important physiological role of HSA is to bind a large variety of ligands (fatty acids, hormones, amino acids, drugs, etc.), and deliver them to the target organs. This remarkable binding property of HSA account for the central role it plays in both the efficacy and the rate of delivery of ligands, and hence has stimulated a great deal of research on the nature of ligand binding sites [10]. However, the binding of ligand depends on its molecular and physical properties and hence all the ligands do not bind at the same site of HSA. The X-ray crystallographic studies reveal that the heart shaped HSA consists of three structurally similar domains (I, II and III), each of which contains two subdomains (A and B) [11], [12]. These subdomains are predominantly helical and extensively cross-linked through several disulfide bridges, with one tryptophan residue (Trp214) in subdomain IIA [13], [14]. It is suggested that the principal regions of ligand binding to HSA are located in hydrophobic cavities in subdomains IIA and IIIA, which are designated as sites I and II, respectively [15], [16].

To understand the pharmacological actions of a particular drug and the relationship of its structure and function, the mode of binding between the drug and the protein under different pH conditions should be studied. Since HSA is known to undergo different pH-dependent conformational transitions it is an ideal candidate for studying protein-drug interaction [17]. At pH 7, HSA assumes the normal form (N) which abruptly changes to a highly charged fast migrating form (F) at pH values less than 4.3, as this form moves “fast” upon gel electrophoresis [18]. The N-F transition involves the unfolding and separation of domain IIIA from the rest of the molecule without significantly affecting the rest of the molecule [19], [20]. Though the pH of blood is generally stable, there is a difference in pH among blood, cerebral blood flow and intracellular and extracellular environments where the drug-HSA interaction takes place. So the transition states of the HSA can affect the binding affinities of the drug, which in turn influence the concentration of the drug in the blood, thereby affecting its biological functions [21], [22]. Moreover, serum albumins are effective in increasing the solubility of hydrophobic drugs in plasma and thereby facilitate their delivery to cell *in vivo* and *in vitro*.

In this study to explore the binding of virstatin to HSA (N, B and F forms), UV absorption spectroscopy, steady-state fluorescence, quenching of tryptophan fluorescence and ITC measurements were carried out. Moreover, far-UV CD, near-UV CD and FT-IR spectroscopy was employed to confirm the conformational changes of the protein upon drug binding. The probable binding site of virstatin to HSA is also predicted from molecular docking studies.

## Materials and Methods

### Materials

Both human serum albumin (free from fatty acid, CALBIOCHEM Cat# 126658) and virstatin (CALBIOCHEM Cat# 677520) were purchased from Merck, Germany and were used without further purification. The stock solution of virstatin was prepared in 100% DMSO. Spectroscopic sample of HSA was prepared by weighing and dissolving the protein in different buffers set at a given pH. For N, B and F conformational states of protein 0.1 M potassium phosphate (pH 7.2 and 9.0) and 10 mM acetic acid/sodium acetate (pH 3.5), respectively were used. The exact concentration of HSA was determined spectrophotometrically using molar extinction coefficient of 35700 M^−1^ cm^−1^ in a UV-1800 Shimadzu Spectrophotometer. All the other chemicals were of analytical grade and used as supplied without further purification.

### UV Spectroscopic Measurements

The UV absorption spectra of the drug along with HSA in the molar ratio of 1∶1 (10 µM) at pH 7.2 were recorded on a UV–1800 Shimadzu spectrophotometer from 700–200 nm. Baseline was corrected using 0.1 M potassium phosphate buffer pH 7.2.

### Fluorescence Measurements and HSA Denaturation

Fluorescence spectra were measured with a Hitachi F–3010 spectrofluorimeter at 25°C with a 1 cm path length quartz cuvette. Both the excitation and emission band passes were kept at 2.5 nm. The excitation wavelength was set at 295 nm to selectively excite the tryptophan residue. Binding of bis-ANS to HSA was measured at 25°C using excitation at 395 nm using slit widths of 5 nm and measuring the emission fluorescence spectra between 420 and 600 nm. Bis-ANS was added to 5 µM of HSA and the changes in ANS fluorescence were followed by measuring the intensity at 495 nm. For energy transfer experiments, an excitation wavelength of 295 nm was used to selectively excite tryptophan residues. Quenching experiment of HSA was performed with successive addition of virstatin and the fluorescence values were corrected for the inner filter effect using the following equation
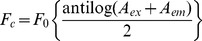
(1)where *F*_0_ is the observed fluorescence, *F*_c_ is the corrected fluorescence, *A*_ex_ and *A*_em_ are the absorbances of the drug at the excitation and emission wavelength.

Urea induced unfolding was used to study the stability of different isomers of HSA (N, B and F) in the absence and presence of virstatin (HSA/virstatin = 1: 1 molar ratio). Stock solution of urea (10 M) was prepared and then aliquots were used to prepare a series of solutions containing different concentrations of urea. The final solution mixture was incubated overnight at room temperature.

### Binding of Virstatin to HSA by ITC

Isothermal Titration Calorimetry allows the measurement of the magnitude of the binding affinity, and the two contributing thermodynamic terms: enthalpy (Δ*H*) and entropy (Δ*S*) changes [23], [24]. The binding of virstatin to the two different conformational forms of HSA (N and B) was studied by ITC, carried out on a VP-ITC (Microcal Inc., Northampton, MA) at 30°C. Protein solutions in different buffers (0.1 M potassium phosphate, pH 7.2 and 9.0, respectively) were dialyzed extensively before injecting. The sample cell (approximately 1.4 mL) was loaded with HSA (conc. 57 µM) and virstatin (conc. 600 µM) was injected into the reaction cell. The titration cell was stirred continuously at 310 rpm, which ensured rapid mixing but did not cause foaming on the protein solution. Titrations were performed to ensure full occupancy of the binding sites and until the titration signal was constant. The calorimetric data were analyzed using the MicroCal Origin 7.0 software provided with the instrument. The enthalpy change for each injection was calculated by integrating the area under the peaks and then subtracted with control titrations. The other thermodynamic parameters were calculated according to the formulas
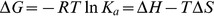
(2)where T is the absolute temperature (303 K) and R = 8.3151 J mol^1^ K^1^.

### Circular Dichroism (CD) Measurements and Thermal Unfolding

CD measurements were carried out with a JASCO spectropolarimeter (model J-800) equipped with a thermoelectrically controlled cell holder under a constant nitrogen flow. Cuvettes with path lengths of 1 mm were employed for far-UV (200–260 nm) and 5 mm for near-UV (240–310 nm) measurements, with HSA concentrations of 10 µM and 1.5 mg/mL, respectively. Each spectrum was the average of three scans. Far-UV CD spectra were collected with a step resolution of 0.1 nm, a scan speed of 50 nm per minute and a bandwidth of 1 nm. All of the CD measurements were carried out at 25°C.

During CD measurements, the DMSO (used as solvent for virstatin) content never exceeded 1.5% (v/v). For the thermal unfolding of HSA in presence and absence of virstatin, far-UV CD spectra were recorded as a function of temperature between 20 and 80°C in steps of 2°C with an equilibration time of 2 min at each temperature. The observed ellipticities were converted into the mean residue ellipticities [*θ*], deg. cm^2^.dmol^−1^] which is given by
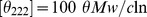
(3)where [*θ_222_*] is the measured ellipticity in degrees, *c* is the protein concentration in mg/mL, *l* is the path length in cm, *Mw* is the molecular weight of HSA and *n* is the number of amino acid residues of HSA. Considering that the unfolding of the HSA is a two-state process between folded *F* and unfolded *U*, the equilibrium constant *K* at any temperature *T* can be written as,
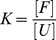
(4)where [*F*] and [*U*] are the concentrations of the folded and unfolded forms, respectively. The equilibrium constant K is related to the Gibbs free energy of unfolding as
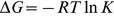
(5)where *R* is the gas constant and *T* is the absolute temperature.

Again the fraction folded at any temperature *α* is given by
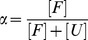
(6)which is 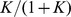 and
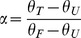
(7)where *θ_T_* is the observed ellipticity at any temperature *T*, *θ_F_* is the ellipticity of the fully folded form and θU is the ellipticity of the unfolded form. The temperature dependence of the secondary structure was estimated from fitted far-UV CD curves by plotting [*θ_222_*] as a function of temperature *T*, using Gibbs-Helmholtz equation
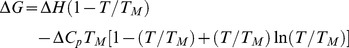
(8)where *T_M_* is the melting temperature, *ΔH* is the change in enthalpy and *ΔC_p_* is the change in specific heat capacity from the folded to the unfolded state.

### Fourier Transform Infrared (FT-IR) Spectroscopy

FT-IR spectra were recorded on a Perkin-Elmer spectrometer equipped with a DTGS KBr detector and a KBr beam splitter. All the spectra were taken via the absorbance mode with constant nitrogen purging. Spectra were obtained at 4 cm^−1^ resolution with 50 scans. Spectra of background were collected and subtracted from the original protein spectra. If not specifically mentioned, all the spectra were collected in the range of 1400–1800 cm^−1^.

### Computational Modeling of the HSA-virstain Complex

Virstatin was docked to the structure of HSA using PATCHDOCK and GOLD [25], [26]. Hydrogen bonding was checked between the protein and the ligand using HBPLUS [27]. The accessible surface area was calculated using NACCESS [28].

## Results and Discussion

### UV Absorption Spectra of HSA in the Presence of Virstatin

**Figure 2:**
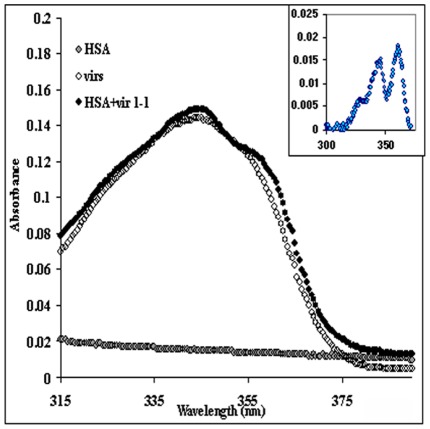
UV absorption spectra of virstatin before and after interacting with HSA in potassium phosphate buffer pH 7.2. The difference spectrum (complex – virstatin) is shown in the inset.

The UV absorption spectra of HSA, virstatin and HSA bound to virstatin were studied at physiological pH (pH 7.2) and are shown in Figure 2. Virstatin has a characteristic peak at 345 nm. However, there was no absorption of HSA alone in this range (400–300 nm), as proteins do not have significant absorbance beyond 300 nm. Upon binding to HSA the wavelength, shape and intensity of the absorption band of the drug changes, with the concomitant appearance of two new peaks at 329 and 360 nm. This can be seen clearly in the difference spectrum (inset, Figure 2).

### Characterization of Different Conformational States of HSA and Analysis of Urea-induced Equilibrium Unfolding Data

The fluorescence emission spectra for different conformers of HSA were recorded in the absence and the presence of virstatin. For native HSA (N and B conformers), the λ_max_ were found to be almost the same (348 nm), whereas for the F form the value of was blue shifted (344 nm) (data not shown). It is likely that at pH 3.5, the environment around the lone Trp becomes more hydrophobic. For all the conformers of HSA (N, B and F) the fluorescence intensity decreases and the spectra are characterized by significant red shift in the presence of 5 M urea indicating the exposure of the sole Trp residue to the aqueous medium [29].

**Figure 3:**
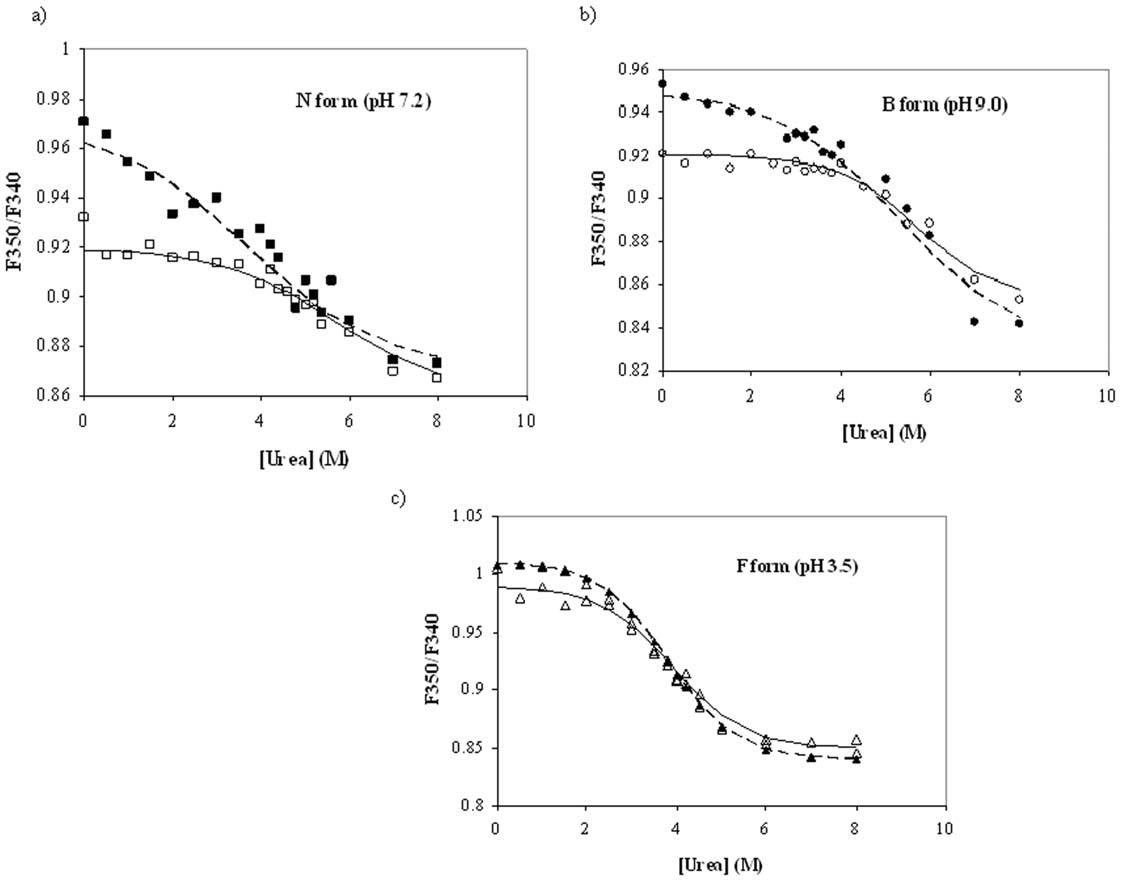
Urea induced unfolding of (a) N, (b) B and (c) F isomers of HSA in the absence (solid symbols) and the presence (open symbols) of virstain. The spectra overlaid along with the best-fit curves assuming a two-state model. F_340_ and F_350_ in the y-axis correspond to the fluorescence intensities at the respective wavelengths.

The effect of virstatin binding on the stability of different isomers of HSA was investigated by urea-induced unfolding at various denaturant concentrations. The equilibrium unfolding was monitored by the measurement of fluorescence emission at 340 nm after exciting the protein at 295 nm. Since HSA contains only one tryptophan residue (Trp214), which resides in domain II, the changes in fluorescence intensity may be ascribed to the conformational changes in this domain. The plots of the ratio of fluorescence emission intensity (F_350_/F_340_) for free HSA (N, B and F states), as well as HSA-virstatin complex against urea concentration yielded sigmoidal transition curves (Figure 3). The unfolding transition curves were analyzed following a simple two-state transition between the folded (F) and the unfolded (U) states. At each urea concentration the observed signal S, representing the ratio of fluorescence emission intensity (F_350_/F_340_) were fitted to a two state equation as shown below
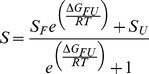
(9)


At each step the free energy change is assumed to be a linear function of concentration of urea. The plots of Δ*G_FU_* (the unfolding free energy) against urea concentration were analyzed using the equation:
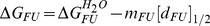
(10)


where *m*_FU_ is the dependency of the Δ*G_FU_* on urea concentration, which is a measure of co-operativity of unfolding and Δ*G_FU_* ^H^_2_^O^ is the free energy change in the absence of denaturant, which is equivalent to the conformational stability of the protein. Dividing Δ*G_FU_* by the slope gives the value for the midpoint of transition, [*d*_FU_]_1/2_.

The result shows that upon binding to the drug (HSA:virstatin in 1∶1 molar ratio) the unfolding free energy Δ*G*_FU_ increases compared to that for the free protein, indicating a stabilizing effect of virstatin on HSA (Table 1). The values of the urea concentration at half-completion of the transition, indicated as [*d_FU_*]_1/2_, were found to be 3.68 M for the free HSA at pH 7.2, which increases to 5.68 M for the HSA-virstatin complex. Similar trends were also observed for the B and F forms of HSA. So irrespective of pH, the binding of virstatin (in 1∶1 molar ratio) makes the protein more stable, as evident from the values of the change in free energy of unfolding as well as the values of the midpoint of transition.

**Table 1:** Unfolding of different conformational isomers for HSA alone and HSA bound to virstatin.

	pH	*G*_FU_ kcal.mol^−1^	*m*_FU_ kcal.mol^−1^.M^−1^	[*d*_FU_]_1/2_ M
HSA (N)	7.2	1.43±0.40	0.39±0.08	3.68
HSA + virstatin	7.2	2.54±0.35	0.45±0.07	5.68
HSA (B)	9.0	2.29±0.35	0.43±0.08	5.29
HSA+virstatin	9.0	3.76±0.60	0.66±0.12	5.71
HSA (F)	3.5	2.93±0.40	0.8±0.09	3.66
HSA + virstatin	3.5	3.03±0.34	0.75±0.10	4.04

Based on data shown in Figure 3.

### Quenching of Tryptophan Fluorescence of HSA Induced by Virstatin and the Calculation of the Binding Parameters

**Figure 4:**
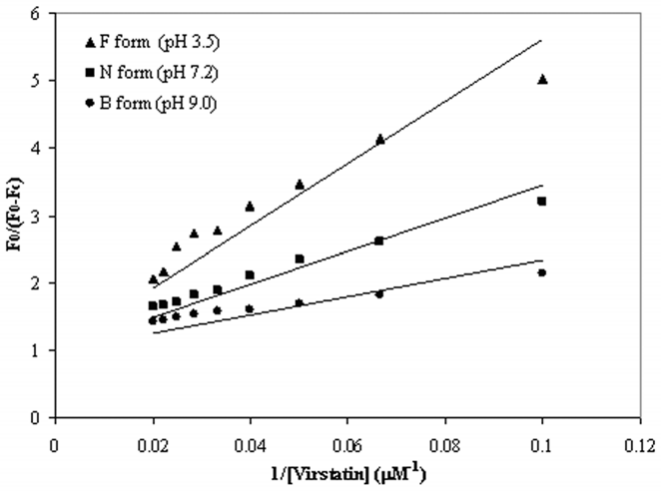
Modified Stern-Volmer plot of the HSA virstatin complex.

The quenching of fluorescence of proteins may be used as an effective tool to derive information about protein-drug interaction [30], [31]. From the Stern-Volmer plot it is evident that the increasing concentration of virstatin caused quenching of Trp fluorescence for all conformational isomers of HSA (Figure S1). As there is a downward curvature in the Stern-Volmer plot, especially for the B form, we used modified Stern-Volmer plot (Figure 4) and obtained the *K_SV_* values [29], which are 1.13×10^5^, 1.76×10^5^ and 0.43×10^5^ M^−1^ for N, B and F conformers of HSA, respectively. The values are in accordance to the ones obtained from ITC (discussed later). Competition studies between warfarin and virstatin were attempted using fluorescence quenching technique to determine the relative sites of their binding to HSA, but did not succeed as both warfarin (excitation at 343 nm) and virstatin (at 310 nm) fluoresce around the same wavelength (emission at 390 nm).

### Bis-ANS Binding Studies

**Figure 5:**
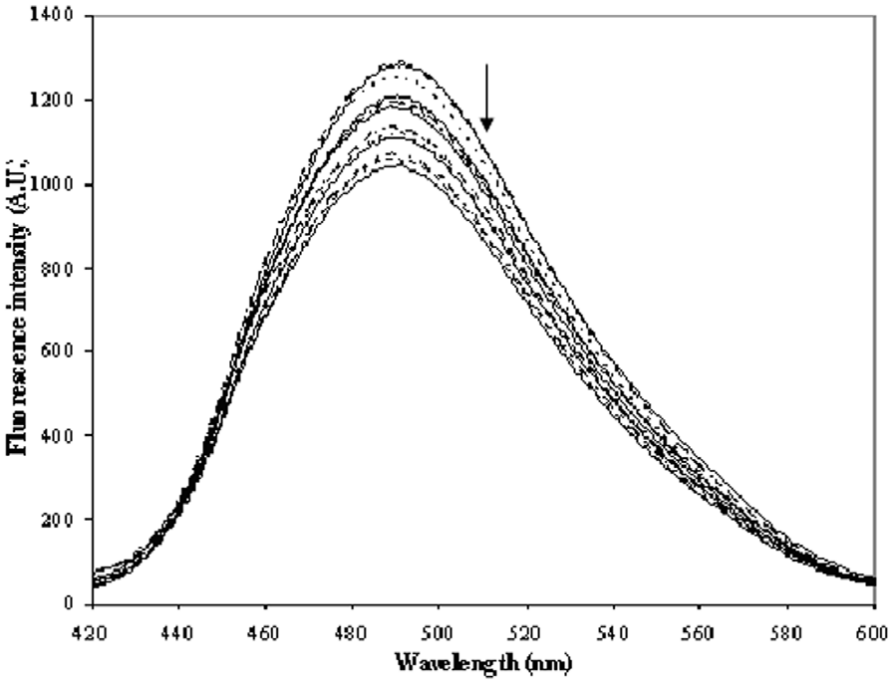
Fluorescence quenching spectra of bis-ANS bound HSA in absence (top) and presence (the arrow indicating curves obtained with increasing concentration) of virstatin in phosphate buffer pH 7.2; [HSA] = 7.5 µM, [bis-ANS] = 5.0 µM and [virstatin] = 0.1 to 5.0 µM (with 10 increments of 0.1 µM, followed by another 5 of 1 µM).

Bis-ANS is widely used as hydrophobic fluorescent probe and hence extensively used to examine the non polar character of protein. A similar molecule, ANS has been shown to bind mainly to HSA subdomain IIIA [32]. We wanted to see if the binding of virstatin has any effect on the fluorescence spectra of bis-ANS bound to HSA. Indeed, there is a decrease in fluorescence intensity of bis-ANS bound HSA on addition of virstatin (Figure 5). It is possible that the changes in conformation of HSA induced by the binding of virstatin (discussed later) cause the quenching of bis-ANS fluorescence.

### Tryptophan Fluorescence Resonance Energy Transfer (FRET) from HSA to Virstatin

**Figure 6:**
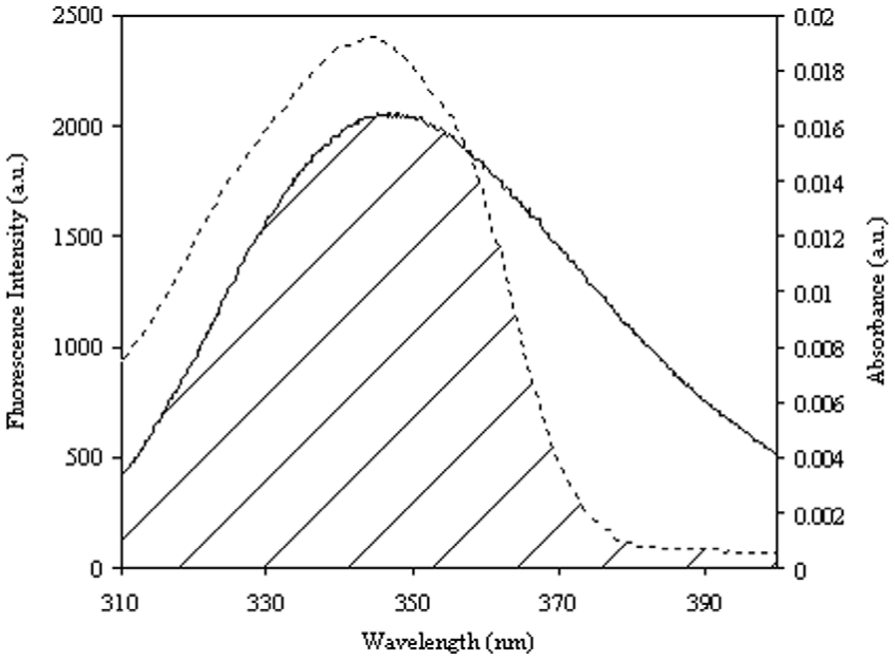
Spectral overlap between fluorescence emission spectrum of HSA (solid line; λ_ex_ 295 nm) and UV absorption spectrum of virstatin (dotted line).

According to Förster’s nonradiative energy transfer theory, the energy transfer is possible when the fluorescence emission spectrum of the donor and UV absorption spectrum of the acceptor have suitable overlap, and the donor and the acceptor are within the characteristic Förster distance [29]. The fluorescence spectra of HSA (7.5 µM) and absorption spectra of virstatin (7.5 µM) were scanned between 300 to 450 nm. The spectral overlap of the donor (W214 of HSA) and acceptor virstatin is shown in Figure 6. The efficiency of energy transfer (*E*) is calculated using the following equation
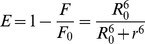
(11)


Where *F* and *F_0_* are the fluorescence intensities of HSA in the presence and absence of virstatin, *r* is the distance between the acceptor and the donor, and *R_0_* is the critical distance when the energy transfer efficiency is 50%. *R_0_* can be calculated using the equation



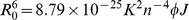
(12)


Where *K* ^2^ is the spatial factor of orientation, *n* is the refractive index of the medium and *φ* is the fluorescence quantum yield of the donor. The overlap integral of the fluorescence emission spectrum of the donor and absorption spectrum of the acceptor, *J* is calculated from the equation
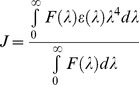
(13)Where *F(*λ*)* is the fluorescence intensity of the donor at wavelength range λ to λ+Δλ which is dimensionless, and ε(λ) is the extinction coefficient of the acceptor at wavelength λ in M^−1^ cm^−1^. In our present study *K^2^*,φ and *n* were taken as 2/3, 0.118 and 1.336 respectively. The value of *r* is 3.05 nm from Eq. 11 using *E* = 0.3. The donor to acceptor distance being within the range of 0.5–2.0 *R_0_* is indicative of an efficient energy transfer from HSA to virstatin. This is further confirmed from the range of *r* which does not exceed the dimensions of HSA (8×8×3 nm) indicating transfer of energy. However the values of *R_0_* and *r* are possibly affected by several factors when calculated by FRET theory and hence must be considered as an apparent measure of the protein-drug binding event [33].

### Isothermal Titration Calorimetry of N and B Isomers of HSA with Virstatin

**Figure 7:**
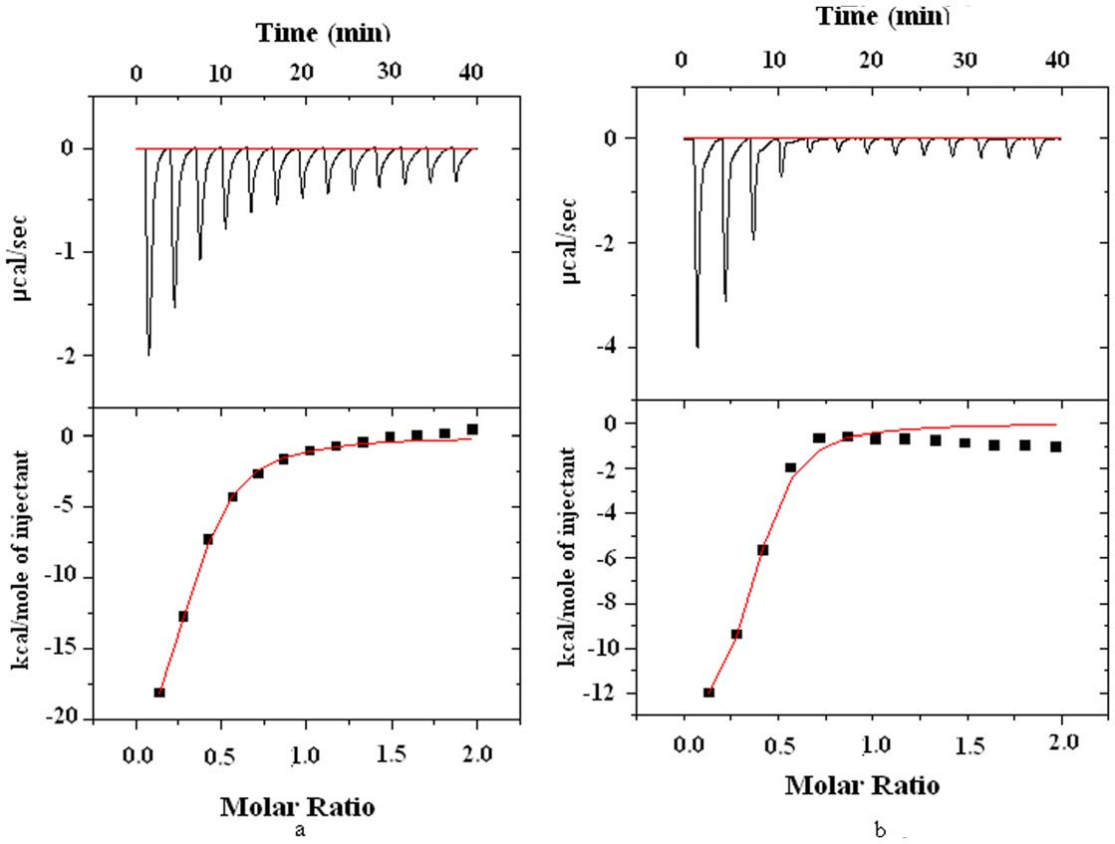
ITC data for the titration of (a) the N and (b) the B forms of HSA with virstatin. Flow of heat with time during the injection of the drug and the heat evolved per mole of added drug for each injection, shown at the top and the bottom, respectively.

In the present study, ITC was used to monitor the binding of virstatin to HSA and to quantify the corresponding thermodynamic parameters. Since ITC can be carried out at higher concentrations of both the protein and ligands, it may complement fluorescence studies, especially for weakly binding probes [34]. It is well reported in literature that the transport function of HSA is controlled through the N-B transition of this protein, which occurs between pH 7.0 and 9.0 [11], [15]. Hence in the present work the N and B conformational isomers of HSA were chosen for binding study with virstatin (Figure 7). The final data were fitted to a one-binding site model and the derived parameters are shown in Table 2. The data revealed that the binding of virstatin to different conformational isomers of HSA (N form and B form) shows a favorable enthalpy change (Δ*H <*0) and an unfavorable entropy change *(*Δ*S <*0). The ligand polarizability in binding to a protein contributes to large negative thermodynamic parameters. Δ*H* and Δ*S* both being negative in this case signifies favorable non-covalent interactions, *viz*., electrostatic, H-bonding and van der Waals between the protein and the drug. From the values of the binding constants (*K_a_*), the stabilities of the N-virstatin and B-virstatin complexes are comparable. With similar binding constants for the N and B isomers the distribution of virstatin in the body is not likely to be affected by the proportion of isomeric forms of HSA.

**Table 2:** Thermodynamic parameters derived from ITC measurements on the binding of virstatin and warfarin with different conformers of HSA.

Thermodynamic parameters	N form	B form
*n* (Virstatin : HSA stoichiometry)	0.29±0.01	0.33±0.02
	[0.96±0.02]	[0.8±0.03]
*K*_a_ (binding constant) M^−1^	(6.09±0.87)×10^5^	(4.47±0.54)×10^5^
	[(3.18±0.54)×10^5^]	[(7.76±1.2)×10^4^]
Δ*H* (enthalpy) kcal mol^−1^	−25.8±0.15	−14.1±0.15
	[−10.8±0.03]	[−15.5±0.09]
*S* (entropy change) cal mol^−1^·K	−58.79	−20.79
	[−10.60]	[−28.75]
*G* (free energy change) kcal mol^−1^	−7.99	−7.80
	[−7.32]	[−6.07]

Values for warfarin are in square brackets.

In order to compare the binding affinities with warfarin the ITC measurements were also carried out to the N and B conformers of HSA (Figure S2 and Table 2). The binding constants for HSA-warfarin complexes were found to be less in comparison to the HSA-virstatin complex, especially for the B isomers. Moreover, there are considerable differences both in the Δ*H* and Δ*S* values between the two ligands, as well as between the two forms of HSA. It is well documented in literature that warfarin binds to subdomain IIA of HSA and shares this binding site with a range of other drugs, *viz*., phenylbutazone, tolbutamide and indomethacin, and thus competes with them for binding [35], [36]. It was also found from docking studies (discussed later) that virstatin shares the same binding site as that of warfarin (site I, subdomain IIA).

### Effect of the Binding of Virstatin on Different Conformations of HSA

To explore various aspects of protein structure and also its interaction with small molecules, CD is one of the strong and sensitive spectroscopic tools [37], [38]. The conformational changes in the secondary structure of HSA have been studied with far- UV CD, in the range of 200–260 nm at pH 7.2, 9.0 and 3.5 (Figure S3). The CD spectra of HSA at physiological pH exhibits two negative bands in the ultraviolet region at 208 nm (π→π * transition) and 222 nm (n→π * transition) which is the characteristic of α-helical protein [39]. The N (pH 7.2), B (pH 9.0) and F (pH 3.5) conformational states of HSA contained 60.2%, 53.4% and 48.5% of α-helix which is in agreement with the values reported by other investigators [40]. The significant loss of helix content for the F form may be due to the disruption of the intra-domain and the inter-domain (II and III) structures at pH 3.5.

**Figure 8:**
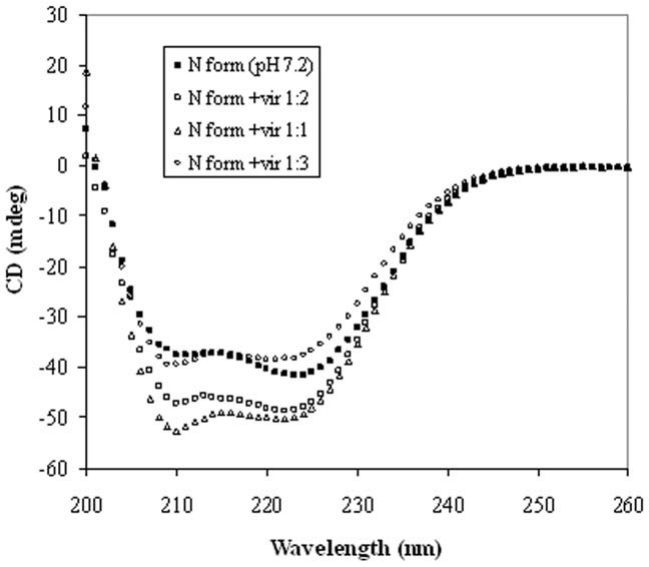
Far-UV CD spectra of the N isomer (pH 7.2) in the presence of virstatin, with HSA/virstatin molar ratios of 1∶1, 1∶2 and 1∶3.

To study the influence of virstatin on the secondary structure of HSA, the far-UV CD spectra were recorded using various molar ratios of drug to protein (0, 1∶1, 2∶1 and 3∶1) (Figure 8). For all conformational states of HSA (N, F and B), there was an increase of α -helical content at the expense of random coil when virstatin binds to protein in 1∶1 molar ratio (Table 3). Further increase in the concentration of virstatin (2∶1 and 3∶1) has a deleterious consequence on the secondary structure (especially, helix) of HSA with a concomitant increase in the percentage of random coil. The perturbation of the secondary structure of HSA in the presence of higher concentration of virstatin has also been noted with other drugs [41], [42].

**Table 3:** Secondary structural content of HSA upon interaction with virstatin in different molar ratios.

Molar ratio (HSA to virstatin)	N form (pH 7.2)			B form (pH 9.0)			F form (pH 3.5)		
	α-helix (%)	β-sheet (%)	Random coil (%)	α –helix (%)	β-sheet (%)	Random coil (%)	α –helix (%)	β-sheet (%)	Random Coil (%)
0	60.2	20.8	19.0	53.4	22.7	21.6	48.5	25.4	24.4
1∶1	63.4	19.3	16.5	64.5	18.7	14.4	64.0	19.5	17.4
1∶2	57.1	21.6	21.3	50.7	20.3	28.5	55.0	22.6	21.3
1∶3	50.4	24.3	24.5	43.6	14.5	39.5	31.2	24.6	44.6

Data were deconvoluted using CDNN software (http://bioinformatik.biochemtech.uni-halle.de/cdnn).

**Figure 9:**
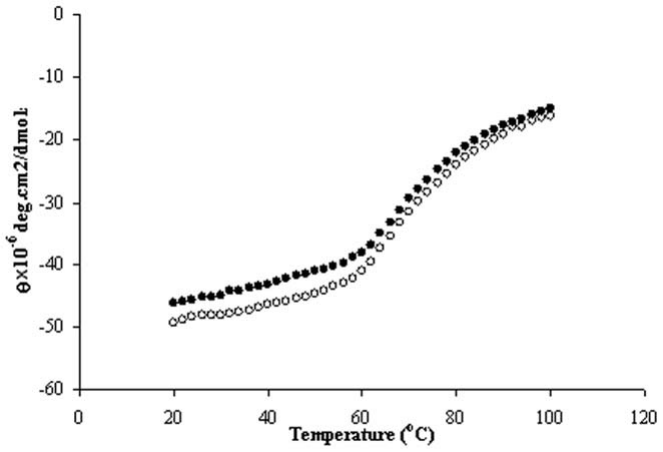
Temperature-induced denaturation of the N conformer of free HSA (solid circle) and in the presence of virstatin (open circle).

Temperature denaturation of protein results from the weakening of interactions, such as hydrogen bonding [43]. In the present study the thermal unfolding of N, B and F conformers of HSA alone, as well as, in the presence of virstain was carried out and the shape of the melting curves conforms to two step transition from the native to the unfolded state (Figure 9). The CD value (*θ_222_*) decreases with the rise in temperature, which may be due to a loss of higher order secondary structure, i.e., α -helix with the concomitant increase of random coil content [44]. This finding is consistent with the characteristics of HSA at higher temperature where the free sulfhydryl group at Cys34 exchanges with other disulfide bridges [45], [46]. The midpoint of the unfolding transition (*T_M_*) determined from sigmoidal fits showed that upon binding to virstatin the value of *T_M_* increased for the N isomer as compared to the free HSA. The values of *T_M_* for N, B and F conformers of HSA were found to be 68.6, 71.2 and 51.0°C, whereas in presence of drug the *T_M_* values were 69.5, 71.9 and 51.7°C, respectively (Figure 9, Figure S4).

### FT-IR Spectra of HSA-virstatin Complexes

**Figure 10:**
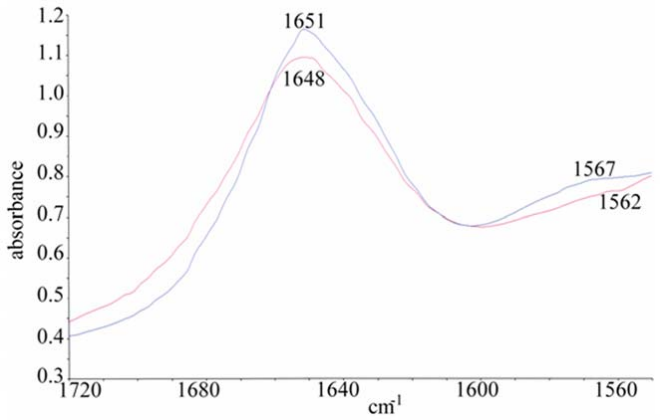
FT- IR spectra of free HSA (blue) and HSA -virstatin complex (red) in D_2_O in the range of 1720 to 1560 cm^−**1**^ **.**

Infrared spectroscopy has been used as a powerful tool to understand the secondary structures of proteins [47]. In the IR region, the frequencies of bands due to the amide I–III vibrations, particularly the amide I band, are sensitive for the prediction of secondary structure of proteins. Hence to further prove the conformational change of HSA induced by the binding of virstatin, we investigated the FT-IR spectra of HSA and the HSA-virstatin complex. The amide I peak position occurs in the region 1600–1700 cm^−1^ and the amide II band at 1548 cm^−1^. In general, the range 1650–1660 cm^−1^ in the amide I band can be attributed to α -helix [48]. In the present study the FT-IR spectra of free HSA as well as virstatin bound HSA was carried out in D_2_O (Figure 10). The characteristic amide I and amide II bands for free HSA were found at 1651 and 1567 cm^−1^, respectively. The peak position of amide I moved from 1651 cm^−1^ to 1648 cm^−1^, and for amide II from 1567 cm^−1^ to 1562 cm^−1^ on complexation with virstatin. This may be due to the change in secondary structure of HSA after interaction with virstatin, resulting in the perturbations of the amide I and amide II vibrational frequencies.

**Figure 11:**
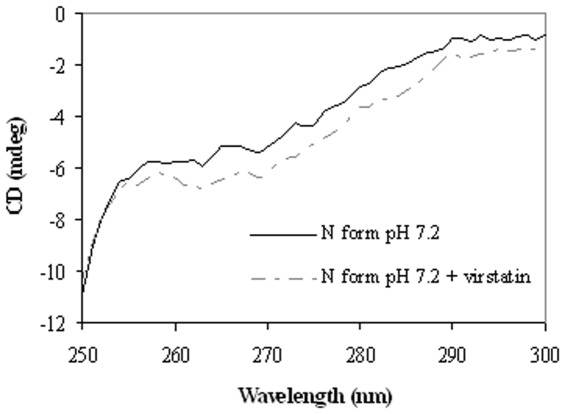
Near-UV CD spectra of the N form of HSA in the absence and the presence of virstatin.

**Figure 12:**
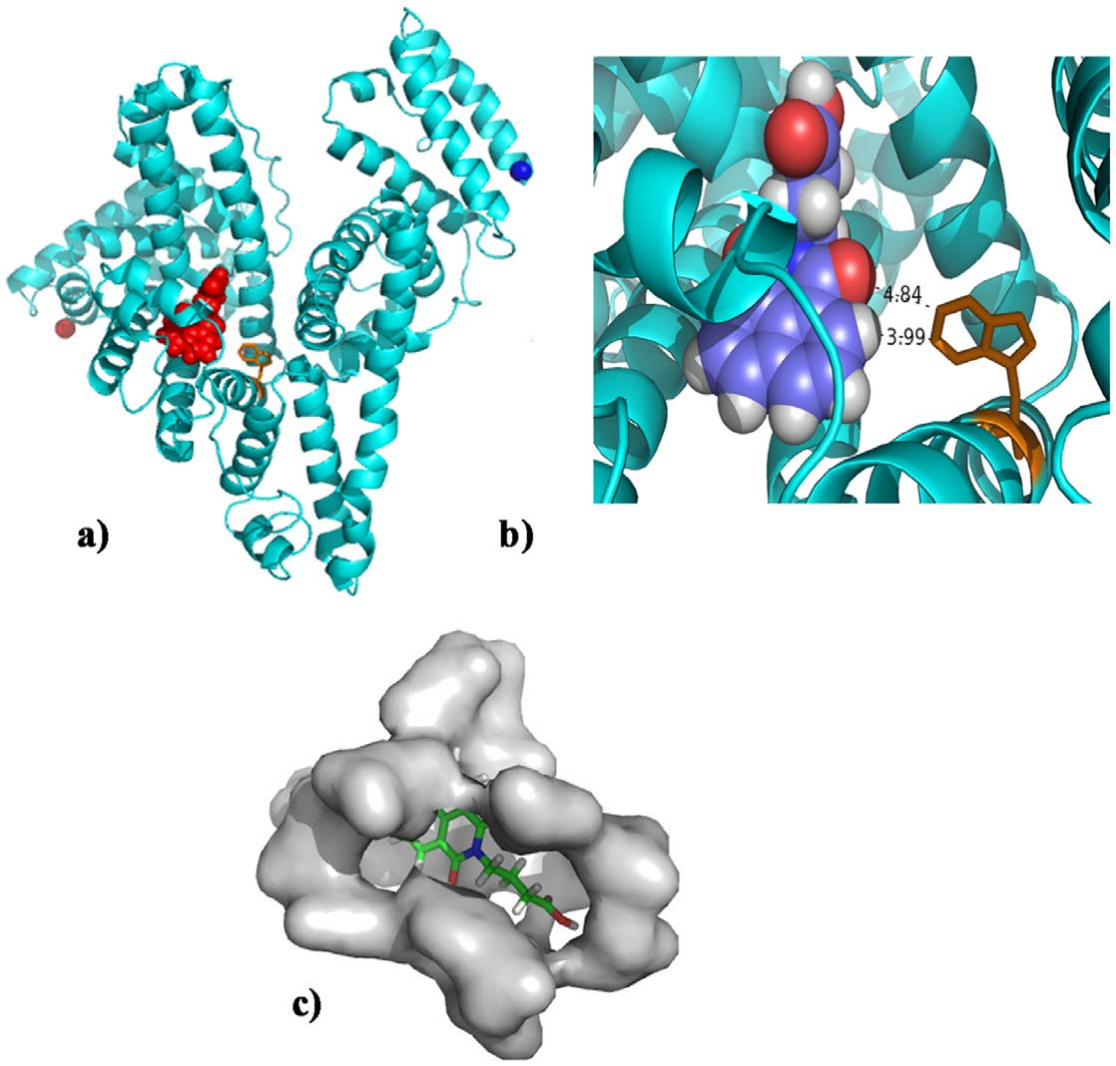
Cartoon representation of HSA with the bound virstatin (red). Trp214 is shown in golden sticks. In (a) the N- and the C-termini of the polypeptide chain are shown as red and blue balls, respectively. (b) Close-up view of virstatin (N is in blue, O in red, H is in grey and the rest are C atoms) with two short contacts with Trp214 shown. (c) The cavity corresponding to site I with bound virstatin. The details of the relative positions of the neighboring residues (Table 4) are shown in Figure S5. The figures are made with PYMOL (http://www.pymol.org).

**Table 4:** Change in accessibilities of residues at binding sites I and II on binding different ligands.

Ligands	Amino acid residue	ΔASA (Å^2^)^b^
Virstatin	Tyr150	15.72
	Glu153	10.69
	Ser192	14.9
	Lys195	17.34
	Lys199	21.69
	Leu219	12.49
	Arg222	13.39
	Leu238	29.68
	Arg257	13.08
	Leu260	11.11
	Ile264	10.07
	Ile290	13.54
	Ala291	34.68
	Glu292	13.57
Warfarin^a^	Leu238	39.03
	Ala291	38.6
	Leu242	35.6
	Trp214	28.93
	Arg257	16.4
	Arg222	14.05
	Leu219	12.25
	Ser287	12.02
	Ile290	11.48
	Phe211	11.26
	Leu260	10.72
Ibuprofen^a^	Leu453	28.22
	Val485	27.55
	Asn391	21.82
	Leu387	19.4
	Ile388	16.85
	Pro384	10.79
	Glu450	10.79

a Values for warfarin binding at Site I and ibuprofen binding at Site II are from [53].

b ΔASA = ASA(isolated) – ASA(complex), where ASA is the accessible surface area of a given residue. Only values >10 Å^2^ are reported.

### Near-UV CD Spectroscopy

Near-UV CD spectroscopy may be employed to detect asymmetry in the environment of aromatic residues and hence is a sensitive tool to measure minor structural perturbations in proteins [49]. Each of the amino acids has characteristic wavelength range, e.g. tryptophan shows fine structures between 290 and 305 nm, while tyrosine between 275 and 282 nm and phenylalanine shows weaker bands around 255 to 270 nm [50]. The near-UV CD spectra were measured in the range of 250 to 300 nm for different isomers of HSA (N, B and F) and also in the presence of virstatin (Figure 11 and Figure S5). For the N conformer (pH 7.2) the appearance of two minima at 263 and 269 nm and shoulders at 273 and 291 nm indicate the characteristics of disulfide and aromatic chromophores, similar to earlier reports. The reduction of CD signal for the B isomer may be due to the fact that during the N-B transition, alteration of tertiary structure of domain I occurred. For the F isomer the signal was further reduced, indicating perturbations around the Trp residue and the disulfide bridges. After binding to virstatin, which itself is an achiral molecule and does not exhibit any CD signal, the near-UV CD spectra of different conformers of HSA showed significant alteration of tertiary structure [51].

### Molecular Docking Study of HSA-virstatin Interaction

In order to understand the efficacy of a drug as a therapeutic agent, it is necessary to explore the binding site of that drug in proteins. HSA contains three predominantly helical structural domains, each of which is made up of two subdomains having specific structural and functional characteristics. The two main binding sites, namely sites I and II (hydrophobic in nature), are located in subdomains IIA and IIIA, respectively. Site I is known as the warfarin binding site, and prefers to bind large heterocyclic and negatively charged compounds, while site II is suitable for small aromatic carboxylic acids [14], [52].

The binding region of virstatin to HSA obtained from both the docking programs, PATCHDOCK and GOLD [25], [26], is very similar and corresponds to the site I. However, while the solution from PATCHDOCK showed no hydrogen bond, the one from GOLD had 3 hydrogen bonds, but with the n-butyric acid side chain folded over to the top of the cyclic structure. With a more extended side chain and an overall greater non-polar contact the solution from PATCHDOCK was considered for further analysis (Figure 12a). Figure 12b shows the contacts between virstatin and tryptophan residue (Trp214) which are within 5 Å. The cavity corresponding to site I with bound virstatin is shown in Figure 12c. There are 14 surrounding residues, seven nonpolar and seven charged; Arg257 is the closest with NE at a distance of 2.95 Å (Figure S6) from O1 of virstatin (the same pair was found to form a hydrogen bond in docked structure obtained from GOLD). Also hydrogen bonding between ligand and Arg257 has reported in other HSA-drug binding studies, indicating a possible electrostatic component in the interaction between virstatin and HSA. The accessibility of the sole tryptophan residue got reduced by 4.4 Å^2^ after docking with virstatin. Apart from Trp214, the accessibility gets reduced for 21 other residues which include all (except Phe211) used for warfarin binding at site I as reported in [53] and none of the residues from site II binding region (Table 4). We also used a recently developed albumin binding prediction webserver (http://albumin.althotas.com) [54], which also predicted the binding site of virstatin to be more similar to the ligands known to bind at site I rather than at II. Table S1 indicates that 96.4% of the surface area of virstatin gets buried on complex formation.

Virstatin has been found to prevent the intestinal colonization of *V. cholerae* [6]. It has also been shown to interact with two *V. cholerae* proteins, ToxT and Ace, associated with the virulence activity of the organism [7], [9]. For the development of the molecule as drug it is important to understand its biodistribution. In this paper the interaction between HSA and virstatin has been studied using various biophysical methods. ITC revealed the binding of virstatin with different conformers (N and B) of HSA. The binding was found to be exothermic, located in site I corresponding to the binding site of warfarin. On binding virstatin there are alterations in the secondary and tertiary structures, as revealed by the far- and the near-UV CD spectroscopy. Results from ITC experiment showed comparable binding affinities for both the isomers. The protein is stabilized, with higher helical content, against urea and thermal unfolding in presence of virstatin in the molar ratio 1∶1; however, a higher concentration of virstatin seems to have a destabilizing effect. The docked binding site of the molecule is compared to other known drug binding sites and correlated with spectroscopic data. The biological importance of this study lies in understanding the interaction of HSA with virstatin, which will be essential for the future designing of virstatin-inspired drugs.

## Supporting Information

Figure S1 Stern-Volmer plot for the quenching of N, B and F isomers of HSA using virstatin. The samples were excited at 295 nm and the emission at 340 nm was measured.
(DOC)

Figure S2 Binding of warfarin with the (a) N and (b) B conformational isomers of HSA.
(DOC)

Figure S3 Far-UV CD spectra of the N, B and F isomers of HSA.
(DOC)

Figure S4 Temperature-induced unfolding of (a) the B and (b) the F conformational isomers of free HSA (solid symbol) and in the presence of virstatin (open symbol).
(DOC)

Figure S5 Near-UV CD spectra of (a) the B and (b) the F conformations of HSA in the absence and the presence of virstatin.
(DOC)

Figure S6 The relative positions of the neighboring residues (the list given in Table 4) of site I of HSA with bound virstatin.
(DOC)

Table S1 Accessible surface area of virstatin before and after complexation.
(DOC)
