# Supplementary material for: Interaction of Virstatin with Human Serum Albumin: Spectroscopic Analysis and Molecular Modeling
Source: PLoS One. 2012 May 23;7(5):e37468. doi: 10.1371/journal.pone.0037468 (PMC3359307; doi:10.1371/journal.pone.0037468)
Supplement: Figure S1 — Stern-Volmer plot for the quenching of N, B and F isomers of HSA using virstatin. The samples were excited at 295 nm and the emission at 340 nm was measured. (DOC) [file pone.0037468.s001.doc]

Figure S1. Stern-Volmer plot for the quenching of N, B and F isomers of HSA using virstatin. The samples were excited at 295 nm and the emission at 340 nm was measured.
