# Supplementary material for: Interaction of Virstatin with Human Serum Albumin: Spectroscopic Analysis and Molecular Modeling
Source: PLoS One. 2012 May 23;7(5):e37468. doi: 10.1371/journal.pone.0037468 (PMC3359307; doi:10.1371/journal.pone.0037468)
Supplement: Figure S2 — Binding of warfarin with the (a) N and (b) B conformational isomers of HSA. (DOC) [file pone.0037468.s002.doc]

Figure S2. Binding of warfarin with the (a) N and (b) B conformational isomers of HSA.


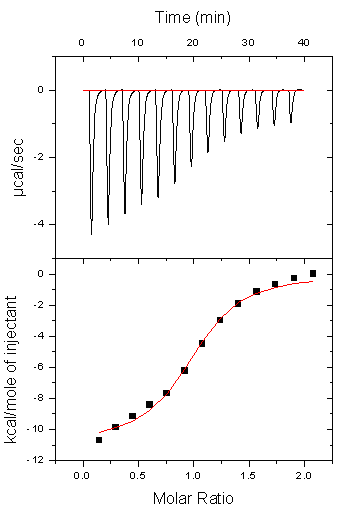


Figure S2(a)


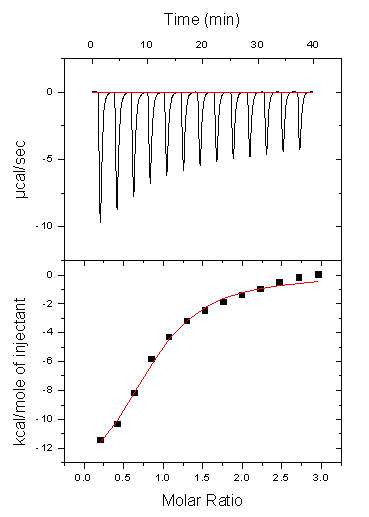


Figure S2(b)
