# Supplementary material for: Interaction of Virstatin with Human Serum Albumin: Spectroscopic Analysis and Molecular Modeling
Source: PLoS One. 2012 May 23;7(5):e37468. doi: 10.1371/journal.pone.0037468 (PMC3359307; doi:10.1371/journal.pone.0037468)
Supplement: Figure S4 — Temperature-induced unfolding of (a) the B and (b) the F conformational isomers of free HSA (solid symbol) and in the presence of virstatin (open symbol). (DOC) [file pone.0037468.s004.doc]

Figure S4. Temperature-induced unfolding of (a) the B and (b) the F conformational isomers of free HSA (solid symbol) and in the presence of virstatin (open symbol)

Figure S4(a)

Figure S4(b)
