# Supplementary material for: Interaction of Virstatin with Human Serum Albumin: Spectroscopic Analysis and Molecular Modeling
Source: PLoS One. 2012 May 23;7(5):e37468. doi: 10.1371/journal.pone.0037468 (PMC3359307; doi:10.1371/journal.pone.0037468)
Supplement: Figure S5 — Near-UV CD spectra of (a) the B and (b) the F conformations of HSA in the absence and the presence of virstatin. (DOC) [file pone.0037468.s005.doc]

Figure S5. Near-UV CD spectra of (a) the B and (b) the F conformations of HSA in the absence and the presence of virstatin.

Figure S5(a)

Figure S5(b)
