# Supplementary material for: Interaction of Virstatin with Human Serum Albumin: Spectroscopic Analysis and Molecular Modeling
Source: PLoS One. 2012 May 23;7(5):e37468. doi: 10.1371/journal.pone.0037468 (PMC3359307; doi:10.1371/journal.pone.0037468)

Figure S6. The relative positions of the neighboring residues (the list given in Table 4) of site I of HSA with bound virstatin.


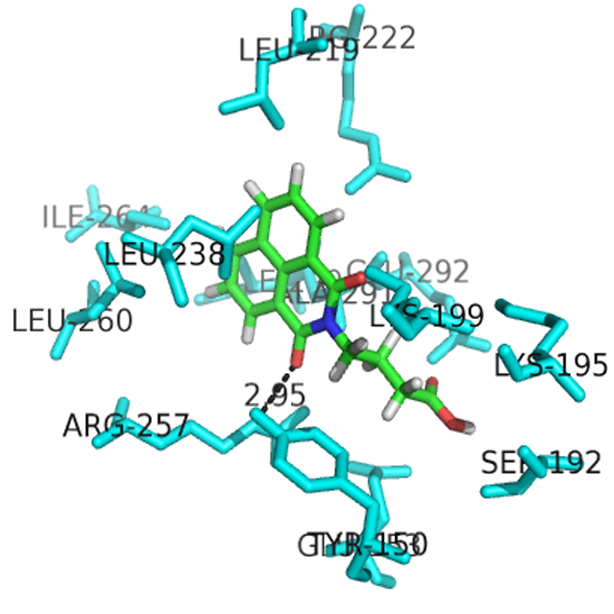

Supplement: Figure S6 — The relative positions of the neighboring residues (the list given in Table 4) of site I of HSA with bound virstatin. (DOC) [file pone.0037468.s006.doc]
