# Supplementary material for: Interaction of Virstatin with Human Serum Albumin: Spectroscopic Analysis and Molecular Modeling
Source: PLoS One. 2012 May 23;7(5):e37468. doi: 10.1371/journal.pone.0037468 (PMC3359307; doi:10.1371/journal.pone.0037468)
Supplement: Table S1 — Accessible surface area of virstatin before and after complexation. (DOC) [file pone.0037468.s007.doc]

Supporting information (Table)

**Table S1. Accessible surface area of virstatin before and after complexation**

| Area of isolated virstatin (Ǻ^2^) | Area of virstatin in complex (Ǻ^2^) |
| --- | --- |
| 448.4 | 16.05 |
